# The Effects of EEG Biofeedback Training on Visual Reaction Time in Judo Athletes

**DOI:** 10.5114/jhk/174272

**Published:** 2023-10-27

**Authors:** Magdalena Prończuk, Grzegorz Trybek, Artur Terbalyan, Jarosław Markowski, Jan Pilch, Michał Krzysztofik, Maciej Kostrzewa, Aleksandra Mostowik, Adam Maszczyk

**Affiliations:** 1 Institute of Sport Sciences, The Jerzy Kukuczka Academy of Physical Education, Katowice, Poland.; 2 Department of Oral Surgery, Pomeranian Medical University in Szczecin, Szczecin, Poland.; 3 Department of Laryngology, Faculty of Medical Sciences, Medical University of Silesia in Katowice, Katowice, Poland.; 4 Department of Physiological and Medical Sciences, The Jerzy Kukuczka Academy of Physical Education, Katowice, Poland.

**Keywords:** Vienna Test System, mental training, decoding device

## Abstract

The aim of the study was to expand the current knowledge on the effects of EEG biofeedback training on the reaction time of judo athletes, as well as to develop an optimal EEG training protocol in terms of the number of sessions and their duration that would significantly improve the reaction time of athletes. The study included 24 male athletes from the national team of the Polish Judo Association. The selected group was randomly divided into two subgroups: experimental and control. The study was conducted in four cycles varying in terms of frequency and duration of neurofeedback (NFB) sessions, both in the control and experimental groups. In the experimental group, each training cycle consisted of 15 sessions, followed by a four-week break. The effects of NFB training on the visual reaction time of judo athletes were evaluated using computerized simple and complex reaction time tests along with selected trials of the Vienna Test System (VTS). Following NFB training according to the theta/beta1 protocol, while maintaining appropriate duration and frequency of individual training sessions, statistically significant improvements in reaction times to visual stimuli of athletes, both in simple and complex tasks, were observed in the experimental group. No such changes were found in the control group. The greatest improvement in reaction times was observed in complex tasks, indicating the high effectiveness of EEG biofeedback training in enhancing this ability.

## Introduction

Since its inception in the 1960s, the concept of biofeedback has found diverse applications in fields such as psychology, medicine, parapsychology, business, and sports to enhance athletes' performance. One of the widely used biofeedback techniques is EEG biofeedback training (Electroencephalography), commonly known as neurofeedback (NFB) (Razran, 1961; Rostami et al., 2012).

NFB represents a more sophisticated form of biofeedback, employing a technological self-regulating stimulation method to restore brain functioning patterns and improve cognitive, emotional, and behavioral performance (Gong et al., 2021; Salimnejad et al., 2019). The analysis of EEG signals, captured using electrodes placed on the subject's scalp, is graphically presented and displayed on a monitor screen, providing feedback on the current changes occurring in their brain. The core principle of EEG biofeedback lies in the assumption that the brain's bioelectrical activity reflects the subject's emotional states and can be controlled and modified through training (Artymiak et al., 2017; Salimnejad et al., 2019).

NFB utilizes measured changes in brain activation to assist athletes in regulating the activity or power of designated EEG frequency bands, offering them real-time activation information (Brito et al., 2022; Paret et al., 2019). EEG has been used in various studies to assess neuronal activity dynamics in the cerebral cortex, with different brain waves recorded, including theta (4–8 Hz), alpha (10–12 Hz), beta (22–15 Hz), and sensorimotor rhythm (12–15 Hz). These waves have been proven useful in understanding the enhancement of cognitive-motor processes (Sherlin et al., 2015). Research conducted among athletes in various sports disciplines such as golf, tennis, archery, soccer, and judo has demonstrated that the development of skills in producing specific cortical activity patterns through NFB training positively affects their efficiency and sports performance (Chung et al., 2021; Landers et al., 1994; Maszczyk et al., 2018, 2020; Saha et al., 2014).

In sports like judo, where visual attention and its contribution to decision-making and motor response planning are crucial for success, a high level of concentration and the ability to quickly react to visual stimuli are particularly important. Thus, it is recommended to focus on improving these skills in the training process of judokas, enhancing their visual processing mechanisms and response time to stimuli (Mirifar et al., 2018).

Reaction time, i.e., the time elapsed from perceiving a stimulus to reacting to it, serves as an indicator for assessing the internal cognitive-motor resources associated with an athlete’s performance. Parsaee et al.’s (2018) and Pourbehbahani et al.’s (2023) studies, which assessed the impact of NFB training on visual and auditory reaction time, revealed that NFB effectively improved brain functions in terms of visual and auditory reaction time.

The objective of the present study was twofold: first, to expand the current knowledge on the effectiveness of EEG biofeedback training to improve the reaction time of judo athletes, and second, to develop an optimal EEG training protocol in terms of the number of sessions and their duration to significantly enhance the reaction time of the studied judokas.

## Methods

### Participants

The study involved 24 male athletes from the national team of the Polish Judo Associationin the southern region of the country, aged between 22 and 25 years. Subsequently, the group was randomly divided into two subgroups: experimental (n = 12) and control (n = 12). All participants received information about the study's objectives and procedures, they were also informed that they could withdraw from the study at any stage. The research was approved by the Bioethics Committee for Scientific Research at the Jerzy Kukuczka Academy of Physical Education, Katowice, Poland and was conducted as part of the grants (N RSA4 04054).

#### Research Procedures

The study was conducted in four cycles, with variations in the frequency and duration of NFB sessions for both the control and experimental groups. In the experimental group, each cycle consisted of 15 training sessions, followed by a four-week break, following the modification of Thompson's training (Thompson and Thompson, 2005). The duration of training sessions was modified from Dupee's training (Dupee et al., 2016), with 10 min and 4 min in successive rounds of the study.

The primary training protocol employed in the experimental group focused on theta/beta1 training, with the objective of improving concentration and attaining a state often referred to as “narrow attention” among athletes. The control group underwent an identical training regimen to the experimental group, with the same cycle, duration, and frequency of NFB training sessions. However, in the control group, instead of implementing the theta/beta1 protocol, an EEG simulation was displayed that was independent of the brain wave patterns generated by athletes.

Before initiating the first training cycle and after completing each subsequent one, simple and complex reaction time tests were conducted in both research groups.

### EEG Biofeedback Training

EEG biofeedback training, also known as NFB training, was conducted using Biograph Infiniti 6.0 software and a 5-channel decoding device (ProComp5). The EEG sensor used in this setup allowed for high-quality signal reception with minimal noise content. The device's quality was confirmed through ISO certification and CE medical certification. Before recording the EEG signal, the impedance levels of the electrodes and interelectrode impedance were checked using a built-in impedance sensor.

To initiate the diagnosis and NFB training, the requirement was to achieve an impedance level below 5 kΩ and a difference of no more than 1 kΩ between electrodes. Each training session in the individual cycle began with a 3-min single-channel EEG diagnosis using three reference connections. During this phase, the participant was instructed to perform specific tasks, including sitting with eyes open for one minute, sitting with eyes closed for one minute, and sitting with eyes open while counting backwards by 7 from 100.

For the diagnosis, the reference electrode was placed on the left ear lobe, grounded on the right, and the active electrode at point Cz, following the international 10–20 system. During NFB training, the active electrode was placed at point C3, which allowed the main training objective to be achieved. This objective was to shape the ability of athletes to maintain an optimal balance between fast (beta) and slow (theta) waves, which are responsible for achieving a state of concentration and focus.

Throughout each NFB session, the percentage of time spent above the threshold was monitored. This measurement served as the primary indicator of the participant's progress and allowed for the optimization of the training difficulty level for each athlete.

During NFB training sessions, real-time feedback was provided to athletes based on their brainwave activity. The main focus was on shaping their ability to enhance the balance between beta and theta waves to achieve an optimal state of concentration and focus. The employed software allowed athletes to visualize their brainwave patterns on a computer screen during training.

NFB training consisted of multiple cycles, and each cycle comprised several sessions. In the experimental group, each cycle included 15 training sessions, followed by a four-week break, as mentioned earlier. The duration of training sessions varied across successive rounds of the study, with sessions lasting 10 min and 4 min alternately, as per the modification of Dupee's training method. Throughout NFB training, researchers closely monitored the percentage of time that athletes spent above the predetermined threshold for beta and theta waves. This measure served as the primary indicator of their progress and allowed researchers to adjust the difficulty level of training for each individual, ensuring that training was tailored to their specific needs and responses.

The goal of NFB training in the experimental group was to increase concentration and achieve what is known as “narrow attention” in athletes. This enhanced the ability to focus and maintain the optimal balance of brainwave activity what could potentially lead to improved athletic performance.

In the control group, NFB training sessions followed the same schedule, duration, and frequency as the experimental group. However, instead of using the theta/beta1 training protocol, the control group underwent an EEG simulation that was independent of their actual brainwave patterns. This setup allowed researchers to assess the specific effects of the theta/beta1 protocol used in the experimental group compared to a non-specific EEG simulation.

Before commencing the first cycle of sessions and after completing each subsequent one, both research groups underwent simple and complex reaction time tests. These tests were conducted to assess any changes in reaction times and cognitive performance resulting from NFB training in both the experimental and control groups.

Overall, EEG biofeedback training with the theta/beta1 protocol aimed to explore the potential benefits of this specific training method for athletes' concentration and focus (Christie and Werthner, 2015; Chung et al., 2021; Landers et al., 1994; Maszczyk et al., 2018, 2020; Saha et al., 2014). The results of the study could have implications for optimizing athletic training programs and enhancing athletes' mental and cognitive capabilities.

### Visual Reaction Time Tests

The study aimed to investigate the impact of NFB training on the visual reaction time of judo athletes. To assess this, computerized simple and complex reaction time tests were conducted, along with selected trials from the Vienna Test System (VTS). The tests were carried out in the morning, ensuring optimal conditions for concentration during the tasks. Each trial was repeated twice at 5-min intervals, and the better of the two measurements was recorded for subsequent analysis.

The computerized simple reaction speed test consisted of athletes pressing a specified key on the keyboard with either their right or left hand as quickly as possible when a bright square appeared on the monitor screen. The complex reaction time task required pressing a key on the keyboard promptly in response to the location of the square that appeared on the screen. Different keys were used for squares in the right or left position and for squares in the central position. In both tests, the signal appeared 10 times, with intervals ranging from 2 to 6 s. The time from the appearance of the stimulus to the key press was measured with high accuracy of 0.001.

For the measurement of simple reaction time to visual stimuli, a reaction speed measuring device (RT) component of the VTS was used. The participant's task was to move their hand as quickly as possible from the “rest key” to the “reaction key” when a yellow LED was lit. The mean reaction time in seconds was then calculated based on the data obtained from the tests.

Complex reaction time was assessed using a decision-making device (DG) integrated into the VTS. In this trial, athletes were tasked with swiftly pressing the corresponding key based on the LED's color that illuminated when the stimulus was presented. The software recorded both correct and incorrect responses, the average reaction time, and the standard deviation of the mean reaction time. The signal was presented 15 times during this testing phase. By analyzing the results from these computerized tests, researchers sought to determine whether NFB training had any significant effect on the visual reaction time of judo athletes.

### Research Cycles

The first research cycle comprised 15 training sessions, conducted every other day, with each session lasting 10 min. In the second research cycle, the training frequency increased, while the duration of each session decreased. Training sessions were held daily, and each session lasted 4 min. In the third research cycle, training sessions took place daily, yet their duration was increased to 10 min. Finally, the fourth research cycle consisted of 4-min training sessions held every other day. All four research cycles were interspersed with a four-week break.

During each training session in all cycles, the percentage of time spent above the threshold was closely monitored. This threshold was adjusted upward for reinforced waves and downward for inhibited waves. Such an adjustment ensured that the level of difficulty of NFB training was optimal and tailored to individual progress.

Overall, the study adopted a progressive approach, modifying the frequency and duration of training sessions across different cycles. By varying these factors, the researchers aimed to assess the effectiveness of NFB training and determine the most suitable training protocol for enhancing athletes' cognitive functions, specifically visual reaction time, and potentially improve their overall performance in judo competitions.

### Statistical Analysis

Descriptive statistics, including arithmetic means, standard deviations, and coefficients of variation, were calculated. The normality of variable distribution was checked using the Shapiro-Wilk test. Additionally, the Levene's test was applied to assess the homogeneity of variance among the variables and determine appropriate statistical tools for further analysis. The test results indicated that all variables had a normal or close-to-normal distribution (*p* > 0.05).

However, it was observed that the groups lacked homogeneity of variance for all variables before training, as determined by the Levene's test. Nevertheless, after training, the values of variables in both groups became homogeneous which was verified using the Levene's test.

To test hypotheses related to differences between the values of individual variables describing intergroup and intragroup relationships, ANOVA was used. The F-statistics and significance levels (*p* < 0.05) were presented. When significant differences were found, Tukey's post-hoc tests were performed for equal sample sizes (N).

The researchers calculated the mean reaction times for both simple and complex reaction time tasks in each group. Changes in reaction time, accuracy, and variability among the athletes were examined. The data analysis aimed to determine if there were any significant differences between the two groups after NFB training.

All the statistical calculations were performed using the Statistica 12.0 (Statsoft) and the Excel package (Microsoft Office 13).

## Results

**Figure 1:**
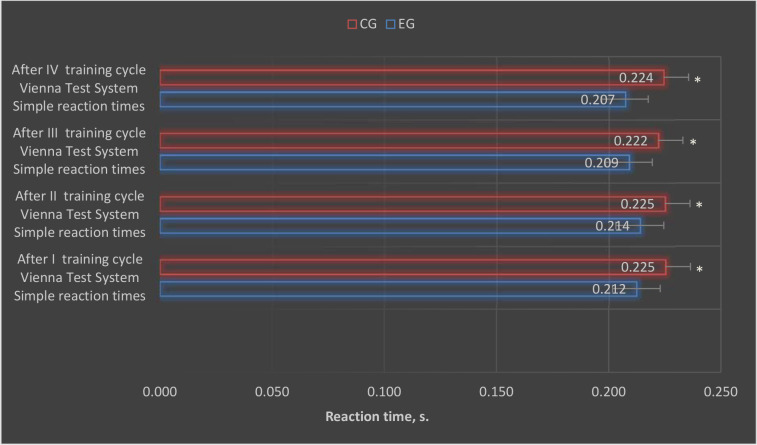
Graphic presentation of simple reaction time results obtained in the experimental and control groups after each EEG biofeedback training cycle. CG: control group; EG: experimental group; *: p < 0.05

**Figure 2:**
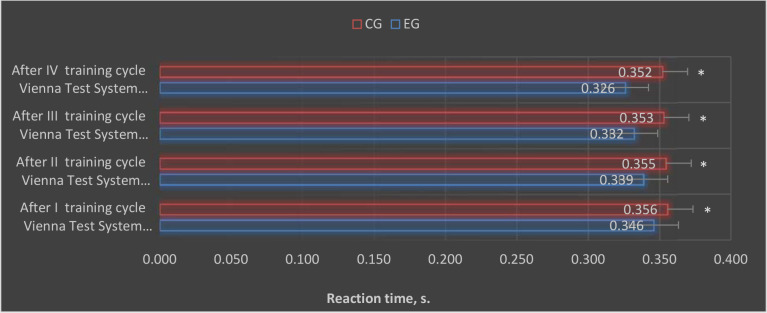
Graphic presentation of complex reaction time results obtained in the experimental and control groups after each EEG biofeedback training cycle. CG: control group; EG: experimental group; *: p < 0.05

After conducting individual training cycles using the EEG biofeedback method, statistically significant differences were observed between the control and the experimental group in the results of simple and complex reaction times in selected trials of the VTS (Figures 1 and 2).

**Figure 3:**
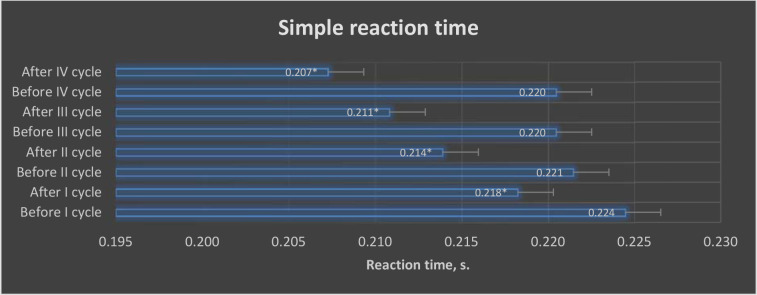
Graphic presentation of differences in simple reaction time results obtained before and after EEG biofeedback training cycles in the experimental group. *: p < 0.05

**Figure 4:**
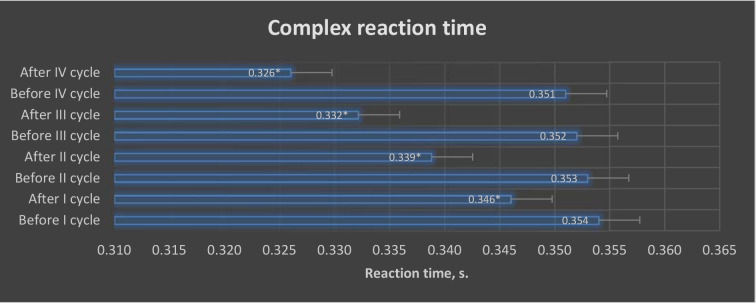
Graphic presentation of differences in complex reaction time results obtained before and after EEG biofeedback training cycles in the experimental group. *: p < 0.05

ANOVA (*p* < 0.05) revealed differences in the values of the investigated variables of simple and complex reaction speeds in selected trials of the VTS before and after the application of individual training cycles using the EEG biofeedback method in the experimental group (Figures 3 and 4).

**Figure 5:**
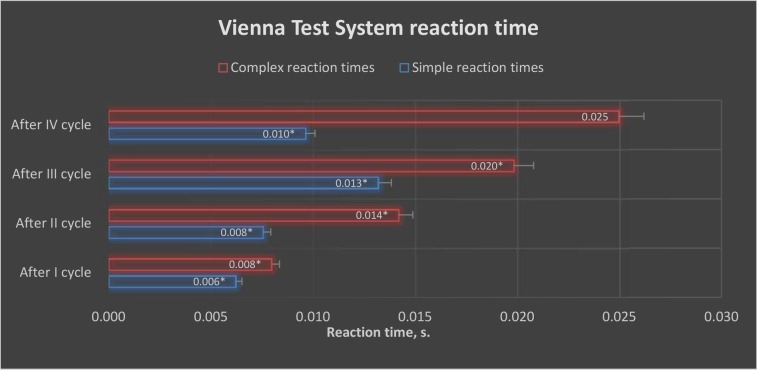
Visualization of improvements in simple and complex reaction times after each cycle of EEG biofeedback training in selected Vienna Test System tasks in the experimental group. *: p < 0.05

Figure 5 presents a graphical visualization of the test-related improvements in simple and complex reaction times before and after the conducted EEG biofeedback training cycles in selected trials of the VTS.

**Figure 6:**
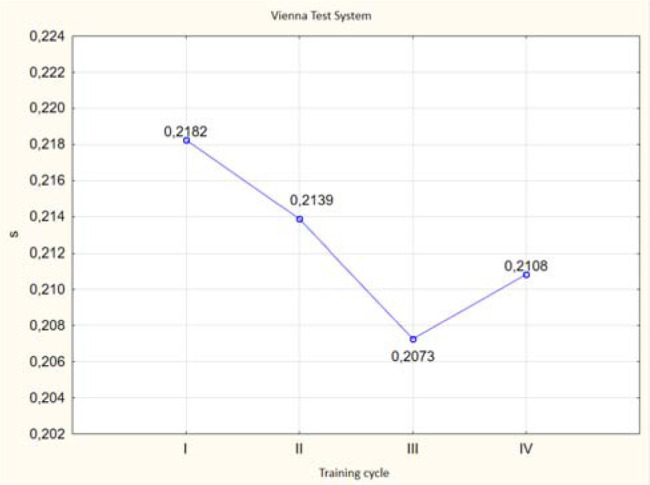
Graphic visualization of differences in simple reaction time test results in selected Vienna Test System tasks between individual cycles of EEG biofeedback training in the experimental group.

**Figure 7:**
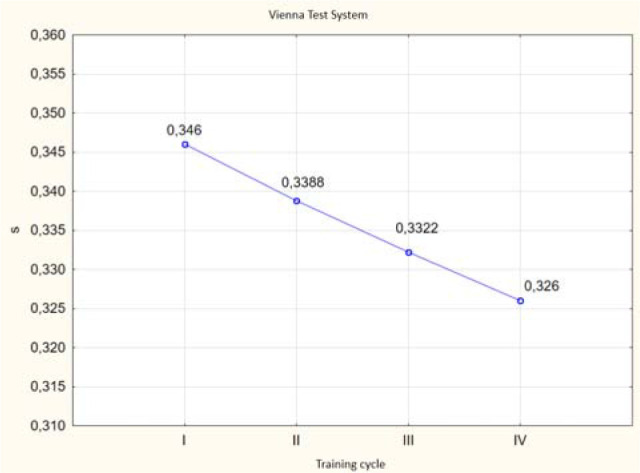
Graphic visualization of differences in complex reaction time test results in selected Vienna Test System tasks between individual cycles of EEG biofeedback training in the experimental group.

The post-hoc Tukey's Honestly Significant Difference (HSD) test indicated significant differences in the obtained simple reaction times in selected trials of the VTS between the third and fourth cycles compared to the first cycle of EEG biofeedback training. However, no significant difference was observed between the fourth and second training cycles. It can be clearly stated that significant improvement in simple reaction time occurred after the third and fourth cycles. Significant differences were noted between the examined variables of complex reaction speed in selected trials of the VTS in relation to the experimental group before the individual NFB training cycles. The analysis of results indicates significant linear differences between the recorded complex reaction times in selected trials of the VTS between the third and fourth cycles compared to the first cycle of NFB training. However, no significant difference was found between the third and second cycles or between the first and second training cycles. It can be concluded that significant improvements in complex reaction time occurred after the third training cycle, and especially after the fourth cycle of NFB training (Figures 6 and 7).

## Discussion

The introduction of modern arousal management techniques in mental training of athletes, such as EEG biofeedback training, and the development of optimal training protocols to enhance athletes' potential, can be useful tools in the field of sports psychology. Prior research suggests that athletes can acquire the ability to produce and sustain particular neural brain activity patterns linked to improved performance through EEG biofeedback training (Blumenstein and Orbach, 2012, 2012a; Lansbergen et al., 2010; Maszczyk et al., 2018). Therefore, the present study aimed to investigate the effects of EEG biofeedback training on the reaction time of judo athletes and to develop an optimal training protocol for improving reaction times. The innovation of the applied NFB training protocol was its implementation in four cycles, with varying duration and frequencies of individual training sessions, ensuring alternative measurement conditions to identify the most favorable configuration. The study showed that athletes in the experimental group achieved a statistically significant improvement in simple and complex reaction times after each training cycle as a result of implementing the theta/beta1 protocol. No such changes were observed in the control group. In another study among judo athletes, the most significant decrease in simple reaction times was attained after the second training cycle, during which training sessions were held every other day for 4 min (Maszczyk et al., 2020). Moreover, in a study involving members of the Canadian National Speed Skating Team, significant improvements in reaction time were observed between the fourth and fifth (last) weeks of training, involving two training sessions per week (Harvey et al., 2011). This confirms previous findings indicating that athletes can learn to generate specific brain neural activity that leads to increased performance (Engelbregt et al., 2016), as well as complements existing analyses on the positive impact of using different forms of biofeedback training on visual perception and the reduction of athletes' reaction times (Christie and Werthner, 2015). The obtained results are also consistent with previous reports suggesting that by strengthening beta1 activity and inhibiting theta waves over the motor cortex, processes related to visual attention are improved. Thus, it may be concluded that NFB training can significantly improve reaction time in athletes.

The results of the present study clearly demonstrate the positive effects of NFB training on the reaction times of judo athletes. The experimental group, which underwent NFB training according to the theta/beta1 protocol, showed significant improvements in reaction times to visual stimuli, both in simple and complex tasks. On the other hand, no such changes were observed in the control group, which did not receive NFB training. These findings support previous research that has shown the potential benefits of EEG biofeedback in enhancing cognitive-motor processes and improving sports performance (Chung et al., 2021; Landers et al., 1994; Maszczyk et al., 2018, 2020; Saha et al., 2014).

Our findings indicate that NFB training can be particularly effective in improving complex reaction times, which are crucial in sports like judo where quick decision-making and response planning are essential for success. This improvement in complex tasks highlights the high effectiveness of EEG biofeedback training in enhancing cognitive abilities related to concentration and focus (Christie and Werthner, 2015; Engelbregt et al., 2016).

The study's contribution to the existing literature is valuable as it provides insights into the practical application of EEG biofeedback training in sports performance enhancement. The development of an optimal training protocol based on EEG measurements offers a structured and systematic approach to improving reaction times in athletes. Such information can be beneficial for coaches, trainers, and sports psychologists working with judo athletes or other sports disciplines where quick reactions and focus are crucial for success.

However, there are certain limitations of the study to be considered. Firstly, the sample size was relatively small, consisting of 24 male judo athletes from a specific region. A larger and more diverse sample would increase the generalizability of the findings. Additionally, the study focused solely on male athletes. Future studies could include female participants to understand potential gender differences in the response to NFB training.

Furthermore, the study did not explore the long-term effects of NFB training beyond the four training cycles. It would be interesting to investigate whether the improvements in reaction times are sustained over time or whether additional training sessions are needed to maintain the obtained benefits.

This study provides valuable insights into the effectiveness of EEG biofeedback training in improving the reaction times of judo athletes. The results suggest that NFB training can be a useful tool in sports performance enhancement, particularly for tasks requiring quick reactions and focused attention. Coaches can use this knowledge to design targeted training protocols for athletes, aiming to improve their cognitive-motor processes and achieve better sports results. However, further research with larger and more diverse samples is needed to validate and extend these findings (Chung et al., 2021; Saha et al., 2014).

Future research in the field of EEG biofeedback training and sports performance could explore several areas of interest. Firstly, as mentioned earlier, investigating the long-term effects of NFB training is essential. A longitudinal study tracking athletes' reaction times over an extended period, even beyond the training cycles used in this study, would provide valuable insights into the sustainability of the obtained improvements. Understanding whether the benefits persist over time or if periodic booster sessions are required for maintenance would help optimize training protocols.

Secondly, it would be beneficial to examine the transferability of the improved reaction times to actual sports performance. While NFB training has been proven effective in enhancing cognitive-motor processes, it is essential to determine whether these improvements translate into real-world situations during judo competitions or other sports events. Observing athletes' reactions in live competitions, as well as their success rates in executing specific moves or strategies, would provide a more comprehensive assessment of the training's practical benefits (Landers et al., 1994; Maszczyk et al., 2018, 2020).

Additionally, expanding the scope of the study to include other sports disciplines would be valuable. Different sports require distinct cognitive and motor skills, and NFB training may have varying effects on athletes from different sports backgrounds. Investigating the impact of EEG biofeedback on athletes in various sports, such as team sports like soccer or individual sports like tennis, could lead to a better understanding of its potential applications across different athletic domains.

Furthermore, exploring the individual differences in response to NFB training would be worthwhile. Not all athletes may show the same degree of improvement in reaction times after training. Factors such as baseline cognitive abilities, training adherence, and motivation could influence the outcomes. Identifying individual factors that contribute to training success could help tailor NFB protocols to suit each athlete's needs, making NFB training more personalized and effective.

In terms of methodology, future studies could consider incorporating control groups that receive other forms of cognitive training or traditional physical training without NFB. Comparing the effects of NFB training to other interventions would help establish its unique contributions and advantages. Additionally, using a double-blind experimental design, where both participants and researchers conducting the tests are unaware of group assignment, would minimize potential biases and enhance the study's internal validity.

Finally, advancing the technology used in EEG biofeedback training could improve its efficacy and practicality. Integrating real-time biofeedback into wearable devices or mobile applications would enable athletes to engage in NFB training outside the laboratory setting, making it more accessible and convenient for regular training sessions.

In conclusion, the study on the effectiveness of EEG biofeedback training in improving the reaction times of judo athletes has provided valuable insights into the potential benefits of this technique in sports performance enhancement. The results suggest that NFB training can be a useful tool in sports psychology for enhancing cognitive-motor processes, particularly for tasks requiring quick reactions and focused attention. The development of an optimal training protocol based on EEG measurements offers a structured and systematic approach to improve reaction times in athletes.

While the findings are promising, further research with larger and more diverse samples is needed to validate and extend these findings. Investigating the long-term effects of NFB training, the transferability of improvements to real-world sports performance, and potential gender differences in the response to training would contribute to a more comprehensive understanding of the technique's benefits. Moreover, exploring individual factors that influence training effectiveness and incorporating control groups with alternative interventions would strengthen the study's conclusions (Maszczyk et al., 2018, 2020).

Overall, the study underscores the potential of EEG biofeedback training as a valuable addition to the toolkit of sports psychology, providing a means to enhance athletes' cognitive abilities and improve their sports performance. As sports continue to become more competitive, the pursuit of innovative and effective training methods like NFB can give athletes a competitive edge and help them reach their full potential. Coaches and sports psychologists can utilize this knowledge to design tailored training programs that optimize cognitive-motor processes and foster improved athletic performance in various sports disciplines, not just judo. By continuously exploring and refining the application of EEG biofeedback training, sports science can continue to evolve and contribute to the success of athletes at all levels.

The results of the study provide valuable insights into the potential benefits of NFB training on cognitive functions, such as visual reaction time, in athletes. It could also contribute to the understanding of how NFB training may be incorporated into athletic training programs to optimize athletes' mental and cognitive abilities, leading to better sports performance. Future studies could build on this research and address any identified limitations to further explore the potential applications of NFB training in sports performance and cognitive enhancement for athletes.
